# Preoperative low muscle mass and malnutrition affect the clinical prognosis of locally advanced gastric cancer patients undergoing radical surgery

**DOI:** 10.3389/fonc.2023.1156359

**Published:** 2023-04-26

**Authors:** Ailing Zhao, Chong Hou, Yingzi Li, Yipin Liu

**Affiliations:** Department of Gastroenterology, Yantai Affiliated Hospital of Binzhou Medical University, Yantai, Shandong, China

**Keywords:** locally advanced gastric cancer, sarcopenia, low muscle mass, malnutrition, prognostic nutritional index, PNIS score, clinical prognosis

## Abstract

**Background:**

Gastric cancer is a common and highly aggressive malignant tumor of the gastrointestinal tract that poses a serious threat to human life and health. As the clinical symptoms of early gastric carcinoma are not obvious, many patients are diagnosed in the middle or late stages. With the advancement of medical technology, gastrectomy has become a safer surgical procedure, but it still has a high recurrence and mortality rate after surgery. The prognosis of gastric cancer patients after surgery is not only related to tumor-related factors (i.e., tumor stage) but the patient’s nutritional status. This study aimed to investigate the effect of preoperative muscle mass combined with the prognostic nutritional index (PNI) on clinical prognosis in locally advanced gastric carcinoma.

**Methods:**

The clinical data of 136 patients with locally advanced gastric carcinoma diagnosed by pathology and undergoing radical gastrectomy were retrospectively reviewed. To analyze the influencing factors of preoperative low muscle mass and its correlation with the prognostic nutritional index. Patients with both low muscle mass and low PNI (≤46.55) were assigned a score of 2, and those with only one or neither of these abnormalities were assigned a score of 1 or 0, respectively, according to the new prognostic score (PNIS). The relationship between PNIS and clinicopathological characteristics was analyzed. Univariate and multivariate analyses were performed to identify risk factors for overall survival (OS).

**Results:**

Low muscle mass was associated with a lower PNI (*P* < 0.01). The optimal cut-off value of PNI was 46.55, the sensitivity was 48%, and the specificity was 97.1%. There were 53 (38.97%), 59 (43.38%), and 24 patients (17.65%) in the PNIS 0, 1, and 2 groups, respectively. A higher PNIS and advanced age were independent risk factors for postoperative complications (*P* < 0.01). The overall survival rate in patients with PNIS 2 score was significantly poorer than in patients with scores of 1 or 0 (3-year OS: 45.8% vs 67.8% vs 92.4%, *P* < 0.001). A Multivariate Cox hazards analysis showed that PNIS 2, depth of tumor invasion, vascular invasion, and postoperative complications were independent predictors of the poor 3-year survival in patients with locally advanced gastric cancer.

**Conclusions:**

The combination of muscle mass and the PNI score system can be used to predict the survival outcome of patients with locally advanced gastric cancer.

## Introduction

Gastric cancer (GC) is a highly aggressive malignant tumor that threatens human life. Although the global incidence and mortality of GC have recently decreased, it is still the third leading cause of cancer-related deaths (1). Because the clinical symptoms of early gastric cancer patients are not obvious, most patients with gastric cancer are in the middle and late stages when diagnosed. In addition, due to the lack of effective and timely treatment, the survival rate of advanced gastric cancer patients is low, the clinical prognosis is poor, and the median survival time is less than 12 months. At present, surgery remains the most important treatment for patients with GC. The postoperative prognosis is not only related to tumor staging but the nutritional parameters and immune status of the patients (2). Sarcopenia is a syndrome that results in systemic and progressive loss of skeletal muscle (3), and affects the postoperative prognosis of patients with GC (4). The EWGSOP2 published a revision of the European Consensus, which amended the definition and diagnosis of sarcopenia to include muscle strength as a critical feature of sarcopenia and to diagnose and identify the manifestations of muscles in the body through the detection of low muscle quantity and mass, to judge its severity (3). Skeletal muscle makes up 40-50% of the body’s composition and is the main storage site for protein. Decreased skeletal muscle mass will affect the cardiopulmonary function, exercise ability, and wound recovery ability of patients with gastric cancer after surgery. The incidence of postoperative complications, such as infections and lower limb thrombosis, will also increase. Muscle mass obtained by abdominal contrast-enhanced computed tomography (CT) can be used as an effective biomarker to assess the nutritional status of cancer patients. Low muscle mass is associated with poor outcomes in patients with multiple tumor types (5, 6). The prognostic nutrition index (PNI) is a test index used to evaluate the nutritional status of patients before surgery. Because it is simple to calculate and easy to obtain, a low PNI is related to the poor prognosis of some solid tumors (7, 8). In addition, hypoproteinemia (9), systemic inflammatory response markers such as the lymphocyte/monocyte ratio (LMR) (10), and other clinical parameters are good predictors of preoperative low muscle mass. Although many studies have investigated the effect of low muscle mass and low PNI on the postoperative survival of patients with locally advanced GC, the combined effect of the two on prognosis needs further research. In this study, we evaluated the relationship between low muscle mass, low PNI, and clinicopathological features, aiming to find a reliable evaluation index for correctly assessing the clinical prognosis of patients with locally advanced GC after surgery.

## Materials and methods

### Patients

The clinical data of 136 patients (102 males and 34 females) who were pathologically diagnosed with locally advanced GC and underwent radical gastrectomy in the Department of Gastrointestinal Surgery of a third-class hospital in Yantai between October 2018 and July 2019 were collected. Inclusion criteria: (1) Patients aged over 18 years with locally advanced GC diagnosed by preoperative pathology and undergoing radical gastrectomy; (2) Abdominal enhanced CT examination was performed within two weeks before surgery, and blood samples were collected without treatment within two days after admission. (3) No neoadjuvant chemotherapy, radiotherapy or interventional therapy were performed before surgery. (4) Complete clinical and follow-up data; reading and writing skills and good language communication ability. Exclusion criteria: (1) Patients who received enteral or parenteral nutrition support within two weeks before surgery; (2) Patients combined with other diseases can cause changes in peripheral blood cells, such as infection or hematological diseases; (3) Unable to cooperate or refuse to participate in the study.

### Observation index

General information (i.e., age, gender, and BMI), past medical histories (i.e., hypertension, diabetes, and coronary heart disease), NRS2002 nutritional risk score, surgical techniques, depth of tumor invasion, lymph node metastasis, tumor location and diameter, histological classification, nerve invasion, serosal invasion, postoperative complications, and survival were collected retrospectively. BMI = weight (Kg) divided by height squared (m^2^), and according to the Chinese Obesity Task Force criteria (11), a BMI <18.5 is classified as a low body mass group. The pathological staging of GC was based on the tumor-node-metastasis (TNM) staging classification (the American Joint Commission on Cancer, 8th edition) (12). The severity of postoperative complications was assessed by the ClavienDindo classification system (13). In this study, postoperative complications were defined as Clavien grade II or higher, severe complications were defined as grade III or IV, and no grade V complications were present. Complications occurring within 30 days after surgery were classified as postoperative complications. The survival time was calculated from the date of surgical resection until death or termination of follow-up, with the last follow-up conducted in August 2022.

### Data collection

This study defined muscle mass with the skeletal muscle index (SMI). To avoid bias and ignore patient information, two experienced radiologists intercepted the third lumbar vertebra section of the abdominal contrast-enhanced computed tomography (after contrast injection) through the Picture Archive and Communication Systems (PACS) tracking system and analyzed the images with Slice-O-Matic software to measure the skeletal muscle area (SMA). Slice-O-Matic software can quantify different tissue types by identifying specific thresholds. The Hounsfield unit (HU) threshold of skeletal muscle ranges from -29 to + 150 (14). Then we calculated SMI by the following formula: SMI (cm^2^/m^2^) = [SMA (cm^2^)]/[Height (m^2^)]. The blood examination data within two days after admission were collected, and the prognostic nutrition index was calculated as (PNI= serum albumin value +5× total number of peripheral blood lymphocytes) (15). The NRS2002 nutritional risk score was used to screen the nutritional risk of hospitalized patients.

### Statistical analysis

Using SPSS 26.0 statistical analysis software, the Youden index was calculated by ROC curve to determine the optimal PNI cut-off value. According to this value, the patients were divided into the high PNI group and low PNI groups. In this study, the lowest quartile was considered as the cut-off point for determining the presence or absence of muscle mass, with SMI < 40.6 cm^2^/m^2^ for men and SMI < 30.5 cm^2^/m^2^ for women as the dividing line. Using the new PNIS prognostic score (16), patients with both low muscle mass and low PNI were scored as 2 points, low muscle mass alone or low PNI alone was scored as 1, and no abnormality was scored as 0. A chi-square test was used for the categorical variables, and logistic regression analysis was used to compare the differences in clinicopathological features among the three groups. A Kaplan-Meier survival curve was drawn to analyze the relationship between low muscle mass, low PNI, PNIS, and postoperative clinical prognosis. Cox proportional hazards model was used to analyze the factors affecting the overall survival of postoperative patients with locally advanced GC. A *P* < 0.05 indicated statistical significance.

## Results

### Patient characteristics

In this study, 102 men and 34 women were enrolled (75% vs. 25%), with a mean age of 62 years (range: 38 to 81 years), and 82 patients were over 60 years. According to the TNM staging, there were 15, 44, 75, and 2 patients in stages I, II, III, and IV (11.03% vs. 32.36% vs. 55.13% vs. 1.48%), respectively.

### ROC curve analysis

**Figure 1:**
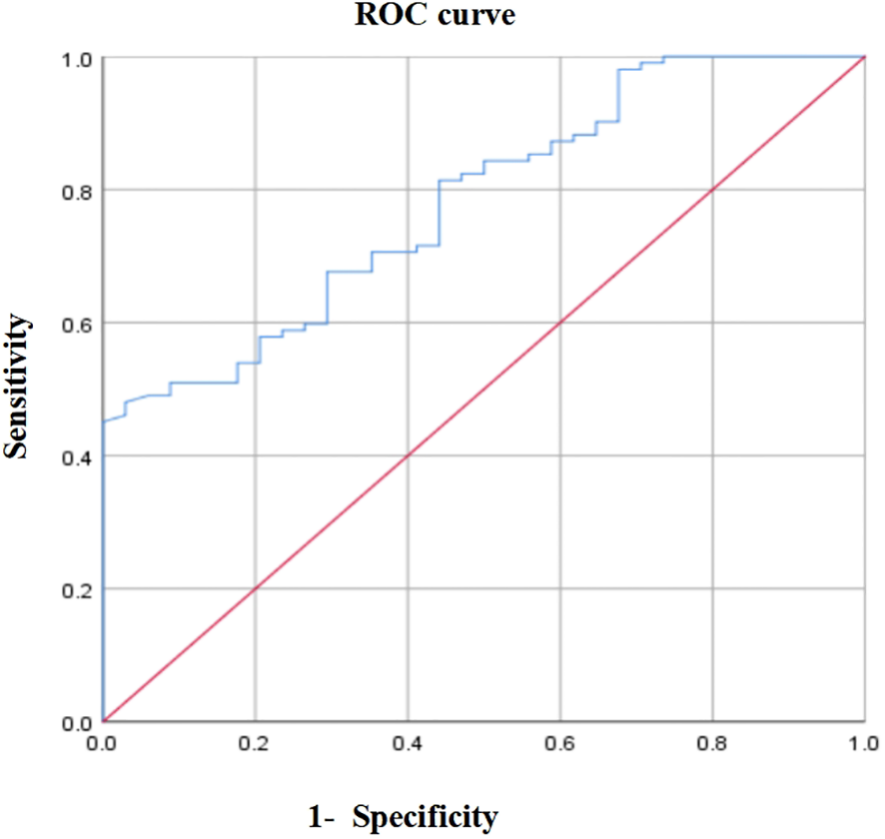
The optimal cut-off value of PNI.

Considering the 3-year overall survival (OS) of patients with locally advanced GC as the endpoint, the area under the ROC curve of PNI was 0.781 (*P* < 0.001, 95% CI: 0.669–0.802). When the PNI=46.55, Youden index was maximal, sensitivity was 48%, and specificity was 97.1% (**Figure 1**). Therefore, the optimal cut-off value of PNI was 46.55. According to this value, 58 (42.65%) and 78 (57.35%) patients were divided into the high and low PNI groups, respectively.

### Correlation between muscle mass and the PNI

The relationship between muscle mass and PNI was weak but statistically significant (r = 0.226, *P* < 0.01). Among the included patients, 29 (21.32%) had low muscle mass, and 78 (57.35%) showed low PNI. The prevalence of low muscle mass was 30.77% and 8.62% in the low and high PNI groups, respectively, and the difference was statistically significant (*P* = 0.009). Univariate analysis showed that age, BMI, NRS 2002 nutritional risk score, PNI, TNM staging, and serum albumin were predictors of low muscle mass (**Table 1**). Binary logistic multivariate analysis showed that age and BMI were independent risk factors for low muscle mass (*P* < 0.05). Significant factors from the above univariate analysis were included in binary Logistic regression analysis, and age and BMI were independent risk factors for low muscle mass (P < 0.05) (**Supplementary Table 1**).

**Table 1:** Predictors of low muscle mass (univariate).

Variable	Low muscle mass (n=29)	High muscle mass (n=107)	Statistical magnitude	*P*-value
Age (yr)			10.338	0.001
≥60	25 (86.21)	57 (53.27)		
<60	4 (13.79)	50 (46.73)		
BMI (kg/m2)			15.664	<0.001
≥18.5	21 (72.41)	104 (97.20)		
<18.5	8 (27.59)	3 (2.80)		
NRS2002 Nutritional Risk Score (points)			5.416	0.020
≥3	25 (86.21)	68 (63.55)		
<3	4 (13.79)	39 (36.45)		
PNI			9.727	0.002
>46.55	5 (17.24)	53 (49.53)		
≤46.55	24 (82.76)	54 (50.47)		
TNM staging			7.590	0.041
I	3 (10.35)	12 (11.22)		
II	4 (13.79)	40 (37.38)		
III	21 (72.41)	54 (50.47)		
IV	1 (3.45)	1 (0.93)		
Serum albumin (g/L)	34.97 ± 3.37	37.37 ± 3.63		0.001

BMI, Body Mass Index; PNI, prognostic nutritional index; TNM, tumor-node-metastasis.

### Correlation between the PNIS and clinicopathological data

We developed a new score (PNIS), with a score of 2 for low muscle mass and low PNI, 1 for only low muscle mass or low PNI, and 0 for patients without these abnormalities. In this study, there were 53 (38.97%), 59 (43.38%), and 24 (17.65%) patients with PNIS 0, 1, and 2 scores, respectively. The relationship between PNIS and the clinicopathological features of patients is shown in **Table 2**. Age, BMI, NRS2002 nutritional risk score, lymph node metastasis, TNM staging, tumor diameter, lymphocyte count, serum albumin, and serum creatinine were included in the ordinal logistic regression. Age, BMI, lymphocyte count, albumin, and serum creatinine were independent risk factors for PNIS (**Table 3**).

**Table 2:** PNIS and clinicopathological features.

Variable	PNIS 0 (n=53)	PNIS 1 (n=59)	PNIS 2 (n=24)	Statistical magnitude	*P*-value
Age (yr)				16.603	<0.001
≥60	22 (41.51)	39 (66.10)	21 (87.5)		
<60	31 (58.49)	20 (33.90)	3 (12.5)		
Sex				2.690	0.261
Male	40 (75.47)	47 (79.66)	15 (62.5)		
Female	13 (24.53)	12 (20.34)	9 (37.5)		
BMI (kg/m^2^)				19.657	<0.001
≥18.5	53 (100)	56 (94.92)	16 (66.67)		
<18.5	0 (0)	3 (5.08)	8 (33.33)		
NRS2002 Nutritional Risk Score (points)				7.731	0.021
≥3	32 (60.38)	39 (66.10)	22 (91.67)		
<3	21 (39.62)	20 (33.90)	2 (8.33)		
Depth of tumor invasion				7.887	0.096
T2	13 (24.53)	9 (15.25)	5 (20.83		
T3	32 (60.38)	36 (61.02)	9 (37.50)		
T4	8 (15.09)	14 (23.73)	10 (41.67)		
Lymph node metastasis				14.905	0.021
N0	17 (32.07)	14 (23.73)	4 (16.67)		
N1	19 (35.85)	9 (15.25)	3 (12.5)		
N2	7 (13.21)	15 (25.43)	7 (29.17)		
N3	10 (18.87)	21 (35.59)	10 (41.66)		
Pathological stage				15.842	0.006
I	8 (15.09)	4 (6.78)	3 (12.5)		
II	23 (43.40)	19 (32.20)	2 (8.33)		
III	21 (39.62)	36 (61.02)	18 (75)		
IV	1 (1.89)	0 (0)	1 (4.17)		
Tumor diameter				6.315	0.043
≥5 cm	21 (39.62)	37 (62.71)	14 (58.33)		
<5 cm	32 (60.38)	22 (37.29)	10 (41.67)		
Nerve invasion				2.332	0.312
Yes	33 (62.26)	30 (50.85)	11 (45.83)		
No	20 (37.74)	29 (49.15)	13 (54.17)		
Serosa invasion				2.318	0.343
Yes	39 (73.58)	50 (84.75)	19 (79.17)		
No	14 (26.42)	9 (15.25)	5 (20.83)		
Vascular invasion				2.380	0.304
Yes	21 (39.62)	28 (47.46)	14 (58.33)		
No	32 (60.38)	31 (52.54)	10 (41.67)		
Serum albumin (g/L)	39.90 ± 2.61	35.27 ± 2.70	34.03 ± 2.67		<0.001
Lymphocyte count (× 10^9^/L)	2.02 ± 0.60	1.61 ± 0.41	1.43 ± 0.36		<0.001
Serum creatinine (umol/L)	65.25 ± 10.95	62.82 ± 12.49	52.69 ± 15.16		<0.001
Postoperative complication				51.807	<0.001
Yes	1 (1.89)	8 (13.56)	18 (75)		
No	52 (98.11)	51 (86.44)	6 (25)		

BMI, Body Mass Index; PNI, prognostic nutritional index; PNIS, new prognostic score.

**Table 3:** PNIS multivariate analysis.

Variable	Regression coefficient	Standard error	WaldX^2^	*P*-value	Exp (B)	95% CI
Age ≥60	1.2793	0.4880	6.8734	0.0087	3.594	1.381-9.353
BMI < 18.5	2.3989	0.8721	7.5670	0.0059	11.011	1.993-60.835
NRS 2002 Nutritional Risk Score ≥3	-0.1751	0.5013	0.1220	0.7269	0.839	0.314-2.242
Lymphatic metastasis						
N0	–	–	–	–	reference	–
N1	-0.1242	0.8073	0.0237	0.8778	0.883	0.182-4.298
N2	0.2759	0.9504	0.0843	0.7716	1.318	0.205-8.489
N3	-0.0647	1.0163	0.0041	0.9492	0.937	0.128-6.870
TNM staging						
I	–	–	–	–	reference	–
II	0.2592	0.9366	0.0766	0.7820	1.296	0.207-8.124
III	0.7129	1.2391	0.3310	0.5650	2.040	0.180-23.138
IV	-1.5883	2.8122	0.3190	0.5722	0.204	0.001-50.580
Tumor diameter (≥5cm)	-0.3560	0.5083	0.4905	0.4837	0.701	0.259-1.897
Lymphocyte count	-2.6843	0.6267	18.3460	<0.0001	0.068	0.020-0.223
Serum albumin	0.00403	0.1025	0.0016	0.9686	0.601	0.500-0.723
Serum creatinine	-0.0458	0.0173	6.9860	0.0082	0.955	0.923-0.988

### Influencing factors of postoperative complications

A total of 27 patients (19.85%) developed postoperative complications in this study. The postoperative complications mainly included pulmonary infection (n = 8), disturbance of gastric emptying (n = 6), infection of incisional wound (n = 4), pleural effusion (n = 3), abdominal infection (n = 2), venous thrombosis of lower extremities (n = 1), abdominal hemorrhage (n = 1), duodenal stump fistula (n = 1), and anastomotic fistula (n = 1). The postoperative complications in patients with PNIS 0, 1, and 2 were 1 case (0.74%), 8 cases (5.88%), and 18 cases (13.24%), respectively, and the differences between the groups were statistically significant (*P* < 0.01). The univariate analysis of postoperative complications showed that there were significant differences in age, gender, NRS2002 nutritional risk score, PNIS, depth of tumor invasion, lymph node metastasis, TNM stage, hemoglobin, lymphocyte count, albumin, and serum creatinine. There were no significant differences in past medical histories (i.e., diabetes, hypertension, coronary heart disease), smoking, drinking, surgical resection, tumor diameter, tumor differentiation, nerve invasion, vascular invasion, serosa invasion, and histological classification (*P* > 0.05). Taking the statistically significant factors in the above univariate analysis as independent variables and the occurrence of postoperative complications as dependent variables, a binary logistic regression analysis showed that age ≥60 and PNIS 2 were independent risk factors for postoperative complications (*P* < 0.01) (**Table 4**).

**Table 4:** Multiple logistic regression analysis of postoperative complications in locally advanced gastric cancer.

Variable	Regression coefficient	Standard error	WaldX^2^	P	Exp (B)	95% CI
Age (≥60)	2.855	1.023	7.793	0.005	17.377	2.341 - 128.99
Sex (Man)	0.0364	0.85553	0.0018	0.9600	1.037	0.194 - 5.544
BMI (<18.5)	-1.0690	1.2471	0.7349	0.3913	0.343	0.030 - 3.956
NRS 2002 Nutritional Risk Score ≥3	1.4598	1.0488	1.9375	0.1639	4.305	0.551 - 33.628
PNIS						
2	3.418	0.844	15.978	<0.001	30.499	5.708-162.952
1	2.1318	1.4987	2.0234	0.1549	8.430	0.447 - 159.051
Depth of tumor invasion						
T2	–	–	–	–	reference	
T3	1.3537	1.8985	0.5084	0.4758	3.872	0.094 - 159.927
T4	1.0502	2.1244	0.2444	0.6211	2.858	0.044 - 183.837
Lymph node metastasis						
N0	–	–	–	–	reference	
N1	1.3630	1.5640	0.7595	0.3835	3.908	0.182-83.805
N2	2.2117	1.9730	1.2566	0.2623	9.131	0.191-436.424
N3	1.1058	2.0766	0.2836	0.5944	0.052	176.942
TNM stage						
I	–	–	–	–	reference	
II	-0.0663	2.7115	0.0006	0.9805	0.936	0.005 - 190.203
III	-0.1628	3.6138	0.0020	0.9641	0.850	0.001 - 999.9
IV	1.7888	6.5932	0.0736	0.7861	5.982	0.001 - 999.9
Hemoglobin	0.00612	0.0143	0.1845	0.6676	1.006	0.978 - 1.035
Lymphocyte count (× 10^9^/L)	0.4517	0.9743	0.2149	0.6430	1.571	0.233 - 10.605
Serum albumin (g/L)	-0.0699	0.1611	0.1885	0.6642	0.932	0.680 - 1.279
Serum creatinine (umol/L)	0.0531	0.0293	3.962	0.0694	1.055	0.996 - 1.117

### Survival outcomes according to muscle mass, the PNI, and PNIS

**Figure 2:**
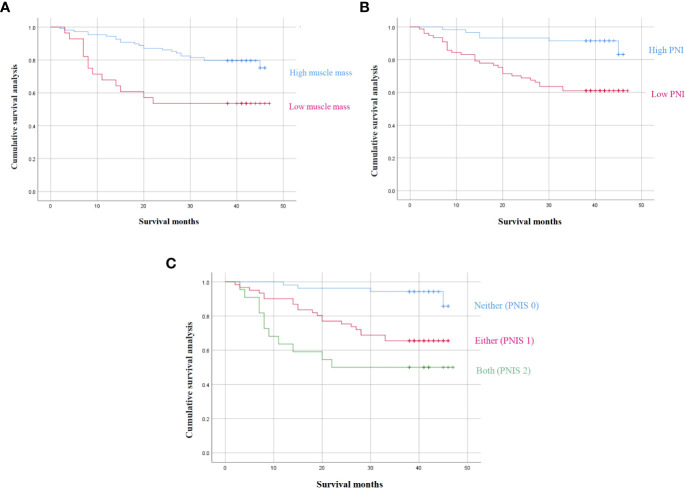
3-year survival outcomes according to **(A)** sarcopenia, **(B)** PNI, and **(C)** the combination of PNI and sarcopenia.

The 3-year OS of patients with low muscle mass was significantly lower than that of the high muscle mass group (*P* < 0.01) (**Figure 2A**). Similarly, the 3-year OS of patients with low PNI was significantly lower than that of patients with a high PNI (*P* < 0.01) (**Figure 2B**). Subsequently, this study evaluated the effect of muscle mass combined with PNI (PNIS) on clinical prognosis. The Kaplan-Meier survival curve showed that the 3-year OS of patients with PNIS 2 was significantly lower than that of PNIS 1 and PNIS 0 (3-year OS: 45.8% vs 67.8% vs 92.4%, *P* < 0.001, **Figure 2C**).

Then, we conducted a univariate study of the 3-year survival. The results showed that age, NRS2002 nutritional risk score, PNIS 2 score, mode of operation, depth of tumor invasion, lymph node metastasis, tumor stage, tumor location, tumor diameter, tumor differentiation, vascular invasion, serosa invasion, and postoperative complications were related to the short-term survival rate of locally advanced GC (*P* < 0.05). A Cox proportional hazard regression model was used to analyze the significant factors in the univariate analysis using the forward method based on partial maximum likelihood estimation. The results showed that high PNIS, depth of tumor invasion, vascular invasion, and postoperative complications were independent risk factors for short-term survival (**Table 5**).

**Table 5:** Cox regression multivariate analysis of postoperative survival rate in patients with locally advanced gastric cancer.

Variable	Regression coefficient	Standard error	WaldX^2^	P	Exp (B)	95% CI
PNIS 1	1.599	0.556	8.275	0.004	4.950	1.665-14.718
PNIS 2	2.569	0.607	17.894	<0.001	13.051	3.969-42.911
Depth of tumor invasion (T4)	-1.438	0.641	5.038	0.025	0.237	0.068-0.833
Combined vascular invasion	-0.913	0.354	6.658	0.010	0.401	0.200-0.803
Combined postoperative complications	-1.312	0.355	13.684	<0.001	0.269	0.134-0.540

## Discussion

The preoperative nutritional status and immune status of patients with GC are correlated with the postoperative and clinical outcomes of patients with malignant tumors (17). Poor nutritional status often leads to decreased immune function, muscle wasting, and poor quality of life, leading to aging and the incidence of diseases. It is thus important to identify prognostic factors to help determine the optimal timing of surgery and postoperative treatment for patients with GC.

Sarcopenia is an important indicator of the nutritional status of the body. The correlation analysis shows that lack of exercise leads to muscle mass decline, which may lead to chronic inflammation and promote tumor growth. In addition, cytokines secreted by muscle cells can also inhibit the proliferation of some tumor cells (18). Sarcopenia can be classified as primary or secondary. In this study, secondary sarcopenia associated with gastric cancer was investigated. Preoperative sarcopenia significantly increases overall postoperative complications (19, 20). A meta-analysis showed a significant association between sarcopenia and poorer overall survival (21). However, there is no international consensus on the best method to measure muscle mass. At present, there are mainly two methods: one is through electrical impedance analysis (BIA) or dual-energy X-ray absorption (DEXA), which directly analyze the body composition and quantify whole body muscle mass, however, at this stage, the above methods are not routine clinical examination, which increases the medical costs. The other is to quantify the cross-sectional area of the skeletal muscle of the third lumbar spine using CT. In clinical practice, almost all patients with GC will undergo abdominal CT examination for a definite diagnosis, staging, or follow-up, and the data are easy to obtain. Studies have confirmed that CT positively correlates with DEXA and BIA (22). Therefore, CT was used to identify and quantify the cross-section of the third lumbar skeletal muscle, and then the slice-O-matic software was used to measure the skeletal muscle area. The results showed that 29 patients had low muscle mass (21.32%), which was consistent with the meta-analysis results (4).

PNI was originally used to evaluate the preoperative immune and nutritional status of patients with GC, which can be obtained by calculating the lymphocyte count and albumin level. It has great advantages in assessing clinical conditions and prognosis, and is an independent predictor of the prognosis of GC (23, 24). However, the mechanism of low PNI affecting the survival rate of patients is unclear, which may be related to the following aspects: PNI reflects the immune level of the body, postoperative patients with GC may have a systemic inflammatory reaction, and the tumor cells themselves have immune escape mechanism, and low immunity leads to a decline in the ability of the body to recognize and kill tumor cells. Patients with low PNI reflect a poor nutritional status, which leads to tissue edema due to hypoproteinemia, increases the possibility of postoperative complications, and may also lead to a different distribution of chemotherapeutic drugs. At present, there is no unified conclusion on the optimal cut-off value of PNI. In this study, the ROC curve was drawn using the 3-year survival rate; when PNI=46.55, the Youden index was the highest. The corresponding sensitivity of predicting the postoperative 3-year survival rate of patients with locally advanced GC was 48%, and the specificity was 97.1%. According to the optimal critical value, the patients were divided into the high PNI (58, 42.65%) and low PNI (78, 57.35%) groups.

This study revealed the correlation between a low PNI and low muscle mass and confirmed the effect of nutritional status on muscle mass. However, up to now, the relationship between postoperative complications and the short-term survival rate of locally advanced GC by combining muscle mass and PNI remains to be further studied. This study aimed to explore a scoring system (PNIS) consisting of muscle mass and PNI, which can be used to predict the short-term prognosis of patients with locally advanced GC undergoing radical resection. According to the PNIS scoring system, the patients were divided into PNIS 0, 1, and 2 groups. A high PNIS score was more likely to lead to short-term complications and a lower short-term survival rate. This study has shown that PNIS is closely related to age, BMI, NRS2002 nutritional risk score, lymph node metastasis, tumor stage, tumor diameter, lymphocyte count, albumin, serum creatinine, and other clinicopathological features, and high PNIS is an independent risk factor for postoperative complications and short-term survival of patients with GC. This result is consistent with the study by Sugawara et al. (16). However, they showed that PNIS 2 is the most effective predictor of poor survival in patients with stage I tumors, but PNIS 2 is not associated with poor survival in patients with stages II and III. The reason for this may lie in the differences in patient distribution. In our study, we excluded patients with early GC. The proportion of patients with stages I, II, III, and IV was 11.03%, 32.36%, 55.13%, and 1.48%, respectively. The results show that the treatment strategy for postoperative patients with locally advanced GC should be based not only on the status of the tumor but also the nutritional status and immune status of the patients. In addition, other factors such as age, depth of tumor invasion, lymph node metastasis, and tumor stage were also influencing factors of postoperative complications and short-term survival rate in patients with locally advanced GC, which is basically consistent with the results reported by Zhuang et al. (25).

It can be seen that there are many factors affecting the prognosis of patients with gastric cancer, and the small sample size in this study leads to relatively few factors included in observation, which is one of the study’s deficiencies. Further, this is a single-center retrospective study; hence there may be subjective bias in the data collection, which cannot achieve the accuracy of prospective study data. In addition, the number of patients in some groups might have been small, limiting the ability of the statistical analyses. Therefore, it is necessary to conduct a follow-up study in a multicenter and using a large sample cohort to confirm the effect of high PNIS on the postoperative prognosis of locally advanced GC.

## Conclusion

Despite limitations, our study confirmed that preoperative low muscle mass with low PNI is associated with poor survival outcomes in patients with locally advanced GC. The new scoring system, PNIS, may help clinicians to identify patients with tumors with poor nutritional status and take early intervention measures to provide individualized treatment and best perioperative management.

## Data availability statement

The raw data supporting the conclusions of this article will be made available by the authors, without undue reservation.

## Ethics statement

This study was reviewed and approved by Yantai Affiliated Hospital of Binzhou Medical University. The committee’s reference number is 20210801018. The patients/participants provided their written informed consent to participate in this study.

## Author contributions

AZ and YPL designed the research. AZ, CH and YZL performed the research. AZ analyzed the data. YPL helped with the statistical analysis. AZ and YPL wrote the paper. All authors read and approved the final manuscript.
