# Supplementary material for: Preoperative low muscle mass and malnutrition affect the clinical prognosis of locally advanced gastric cancer patients undergoing radical surgery
Source: Front Oncol. 2023 Apr 26;13:1156359. doi: 10.3389/fonc.2023.1156359 (PMC10171366; doi:10.3389/fonc.2023.1156359)
Supplement: Supplementary file 1 [file DataSheet_1.zip › Revised-Supplementary Material/Supplementary Table 1.doc]

**Supplement Table 1**.**** Predictors of low muscle mass (Multivariate).

| Variable | Regression coefficient | Standard error | WaldX^2^ | *P*-value | Exp (B) | 95% CI |
| --- | --- | --- | --- | --- | --- | --- |
| Age ≥60 | 1.4676 | 0.6437 | 5.1981 | 0.0226 | 4.339 | 1.229 - 15.320 |
| BMI ＜ 18.5 | 2.0817 | 0.8324 | 6.2544 | 0.0124 | 8.018 | 1.569-40.984 |
| NRS 2002 Nutritional Risk Score ≥3 | 0.8472 | 0.6312 | 1.8015 | 0.1795 | 2.333 | 0.024 - 0.637 |
| PNI≤46.5 | 0.7351 | 0.7554 | 0.9469 | 0.3305 | 2.086 | 0.474 - 9.168 |
| TNM staging |  |  |  |  |  |  |
| I | - | - | - | - | reference |  |
| II | -0.224 | 1.0202 | 0.0475 | 0.8274 | 0.801 | 0.108-5.913 |
| III | 0.8337 | 0.8998 | 0.8585 | 0.3542 | 2.302 | 0.395-13.427 |
| IV | 1.7033 | 2.8983 | 0.3454 | 0.5567 | 5.492 | 0.019-999.999 |
| Serum albumin | 0.00403 | 0.1025 | 0.0016 | 0.9686 | 1.004 | 0.821-1.227 |
